# Oncotarget: Past, Present and Future: Trends in the publishing industry

**DOI:** 10.18632/oncotarget.28852

**Published:** 2026-04-08

**Authors:** Scientific Integrity Office at Oncotarget

The mission of Oncotarget is to maximize research impact through insightful peer-review, eliminate borders between specialties by linking different fields of oncology and biomedical science, and to foster the application of basic and clinical science. This mission cannot be accomplished without following strong ethical standards. Scientific integrity is a crucial component of scholarly publishing. At Oncotarget, a growing industry of digital technologies, tools and ideas is continuously integrated to support a forward- looking and robust scientific integrity process.

**I.** At Oncotarget, we uphold a rigorous scientific integrity process and are committed to continuously updating our technologies and tools to meet the highest scientific standards. Our scientific integrity principles are outlined on our Scientific Integrity page.

On that page, we provide data reflecting the number of confirmed corrections and retractions (both published and pending, current as of August 2024-January 2025) across all years of Oncotarget’s publishing history. The data will be updated in 2026 to reflect 2025’s numbers.

The statistics are based not only on our internal records but also show independent data from Argos, Scitility, a scientific integrity tool that flags the research papers with potential issues, categorized as low-, medium-, or high-risk. Scitility estimates that high-risk articles are approximately 79 times more likely to be retracted. Impact Journals is subscribed to the tool. Our internal and external data sources show consistent results. To the best of our knowledge, Oncotarget is among the few journals that publicly publish such statistics.

**Figure 1:**
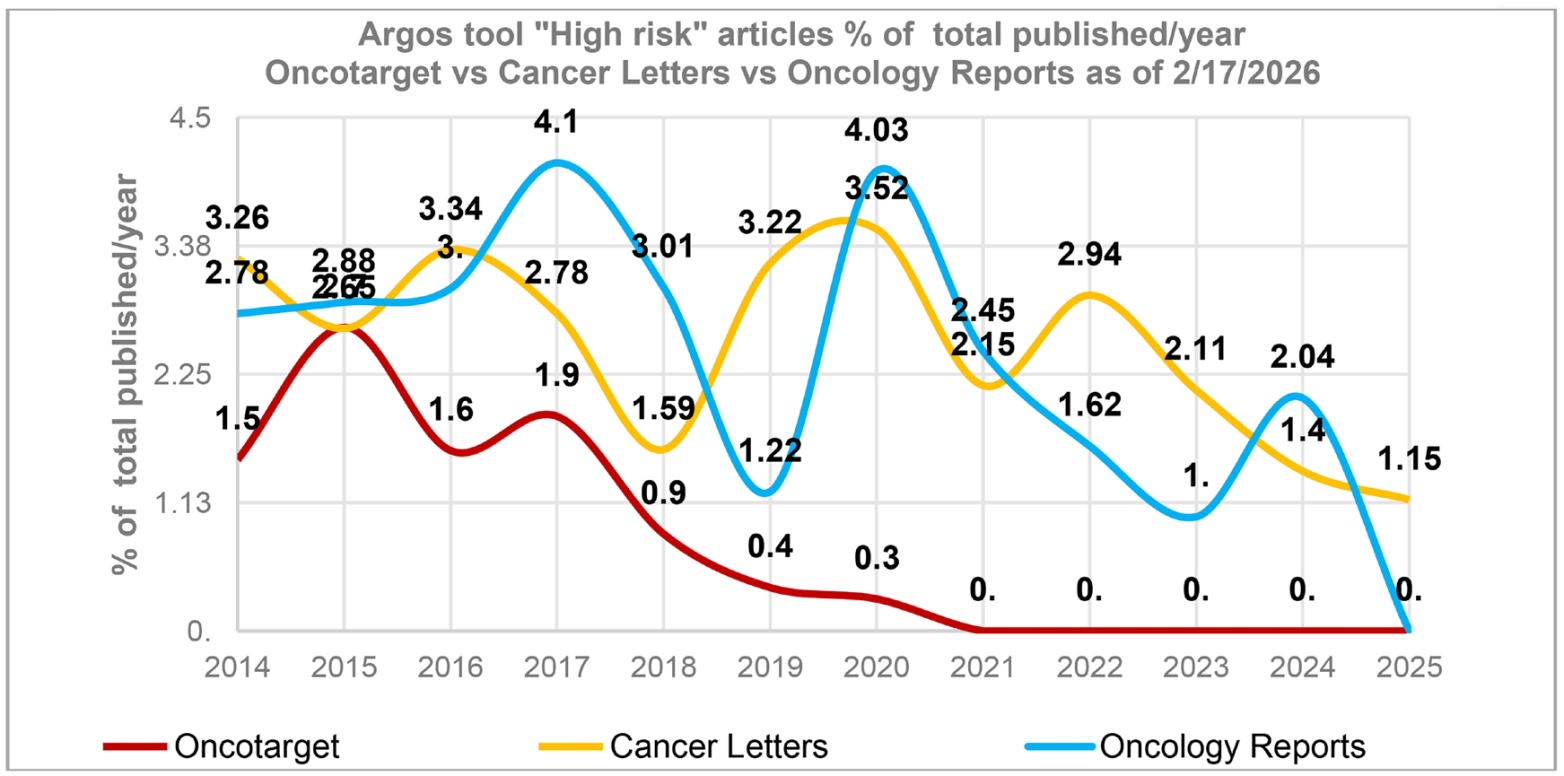
A spike in high-risk articles in 2015–2017. The percentage of “High risk” articles identified by the tool “Argos” (as of 2/17/2026) in relation to the total number of articles published in a journal during a specific year. This percentage is influenced by the journal’s publication volume, with journals publishing a larger number of articles typically having a smaller percentage. The average annual article counts for 2014–2025 for Oncotarget - 2,240 articles/year, Cancer Letters - 493 articles/year and Oncology Reports – 539 articles/year.

The data reveals that the highest number of papers with potential issues occurred between 2015–2017 when, it is important to note, Oncotarget published its highest volume of articles. This trend is not unique to Oncotarget. According to Argos data, other journals, for example, Cancer Letters, Oncology Reports, to name a few, also demonstrated a spike in high-risk papers during the same time period (Figure 1). In addition, a Scitility/Argos analysis of more than 400 journals revealed a slow but steady increase in the number of retracted articles published between 2014 and 2017.

**II**. We believe this trend reflects the changes in the scientific and publishing landscape at that time. While analyzing those shifts is beyond the scope of this article, it is evident that the publishing industry was unprepared for the evolving changes.

One major outcome of addressing these challenges has been the development of tools for image integrity analysis. We first began using internally developed image-checking tools in November 2020, which noticeably reduced the number of the papers with problematic images (see the graph on Oncotarget Scientific Integrity page). Then, we subscribed to the ImageTwin, a highly popular and effective tool as soon as its fully functional version became publicly available. After testing ImageTwin in December 2022, we started using it in January 2023.

Impact Journals also created a dedicated Image Forensics team that analyzes both new submissions and cases involving potential corrections or retractions, working closely with the Scientific Integrity Office.

As a result of our efforts, as of December 31, 2025, there have been zero (0) potential retractions in 2020–2025 (Please see more details below).

Our experience highlights the critical importance of image forensics tools in maintaining scientific integrity. As noted earlier, advanced image-checking tools have only recently become widely available and did not exist prior to 2020. Without such tools, particularly ImageTwin, it was extremely challenging for journals to check images effectively. Cross-examination of image data across different journals was virtually impossible. ImageTwin is a highly popular tool, which is now used by the PubPeer community to evaluate past (published in the pre-tool era) and present publications. **Therefore, we assert that any discussion of past scientific integrity issues should include a retrospective analysis of the publishing industry’s historical limitations.**

We are also investing considerable efforts into correcting the records published in the past.

All internal data presented in this Editorial are as current as of December 31, 2025 and based on an analysis of 23,790 research articles (PubMed). Only research articles that are eligible for the image forensics check (IF) were included in this assessment.

As of December 31, 2025, the total number of *published retractions* across all years is 122, with the majority of original papers published in 2016 (40) and 2017 (62), aligning with the period between 2015 and 2017, as previously noted (please refer to Figure 1 and notes above).

This is how the published retractions are distributed by years:

Advance publications-2;

2010-0;

2011-1;

2012-0;

2013-0;

2014-3;

2015-5;

2016-40;

2017-62;

2018-8;

2019-1;

2020-2025 - 0

We also maintain a list of pending retractions. The term “*pending retraction*” refers to cases where the Scientific Integrity Office has confirmed that data are compromised and is in the process of preparing the manuscript for retraction in coordination with the authors and relevant officials. At present, our pending retractions list includes 60 papers, comprising papers where:

- Authors have submitted retraction requests
- Universities or other institutions have raised inquiries
- Preliminary investigations have confirmed concerns raised by readers.

This is how the pending retractions are distributed by years:

2010-2014- (0);

2015-13;

2016-17;

2017-27;

2018-3;

2019-2025- 0

As of December 31, 2025, there are no pending retractions beyond 2018.

We are planning to publish all 60 pending retractions in 2026.

Based on the current data on retractions and pending retractions, as well as the number of published research articles eligible for IF analysis, the below retraction rates were calculated for different time periods:

**Table:** 

Year	Retraction rate %	Eligible articles	Retractions
2010–2014	0.28	1403	4
2015–2017	0.9	18394	166
2018–2019	0.37	3236	12
2020–2025	0	757	0

Moreover, our internal records are consistent with Argos tool data. According to Argos, as of March 5, 2026, Oncotarget has only two high-risk papers, PII 27804 and PII 27622, published in 2020 (both of which were checked by our Scientific Integrity Office and are not under consideration for retraction), and no high-risk papers have been identified after 2020.

We also maintain a list of potential retractions. The term “*potential retraction*” refers to cases where the journal has received information from various sources suggesting the data may be compromised, but such information has not yet been confirmed. As of December 31, 2025 this list contains 250 papers published between 2011 and 2019. Our internal records align with the reports received from PubPeer associated members, E. Bik and D. Sholto, in April 2025. Their reports also do not include any potential retractions beyond 2019. We are actively working on the potential cases and will publish the results as well as an updated retraction rate for the corresponding time periods. Any new potential cases dated after December 31, 2025 will also be included.

Image forensics tools play a critical role in investigating papers with potential integrity issues. Nearly two-thirds of all retractions were issued between 2023 and 2025, when we started to actively use image forensic tools on a routine basis. The Scientific Integrity Office is actively working to publish pending retractions as quickly as possible.

**III.** As noted above, image forensic tools have permanently changed the landscape of scholarly publishing. **Argos data provide a good opportunity to obtain an objective picture across different journals in the post-tools era.**

**Figure 2:**
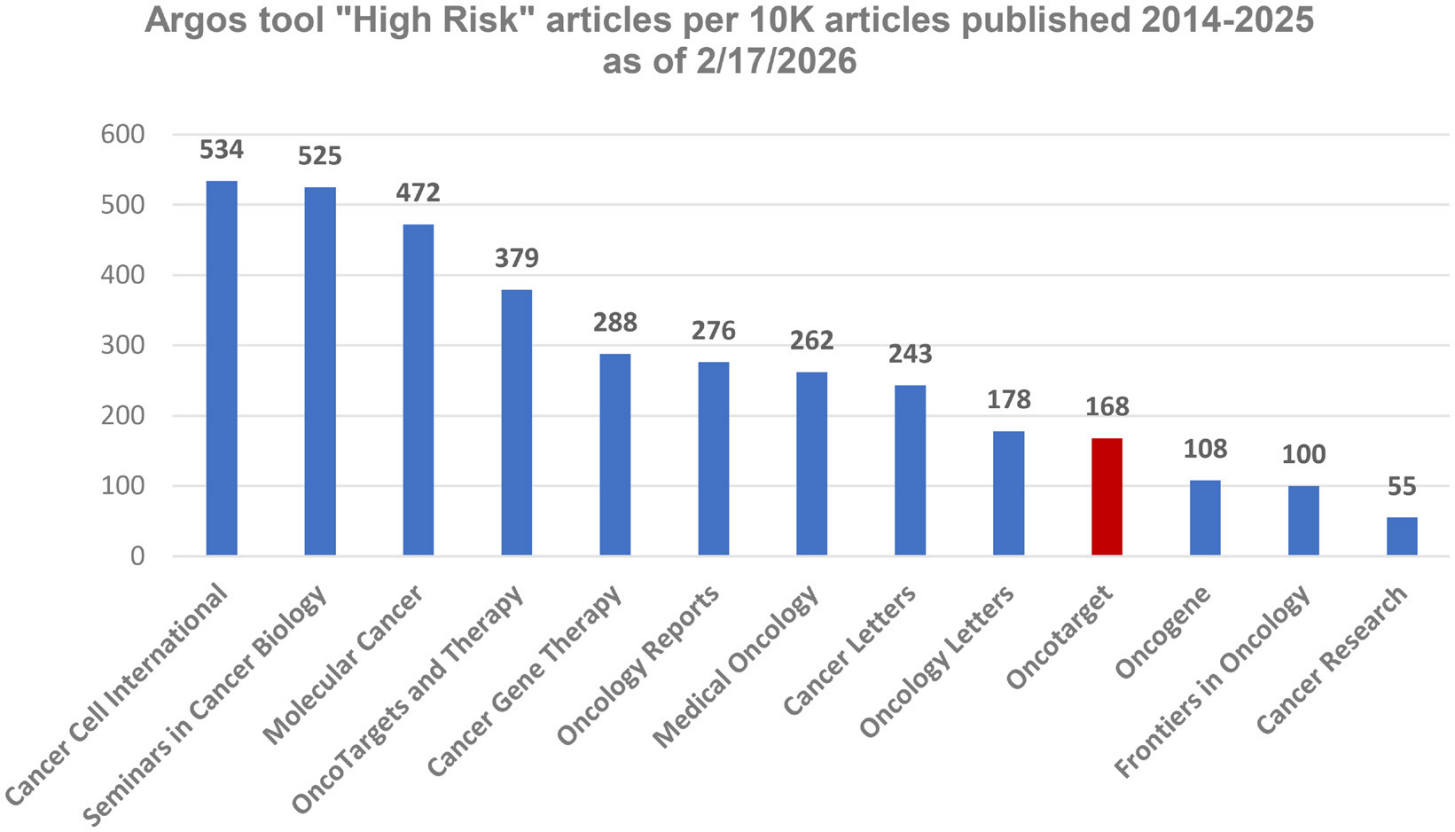
The number of “High risk” articles per 10,000 articles published in oncology-related journals (indexed in WOS are in blue) between 2014 and 2025 (as of 02.17.2026).

Our analysis of 13 oncology-related journals reports the number of high-risk papers normalized per 10,000 published articles (Figure 2). The Journals indexed on Web of Science (WoS) are shown in blue. Oncotarget, shown in red, has not been included in the WoS Core Collection since January 2018. At the end of 2020, Oncotarget submitted an application for reevaluation in accordance with the WoS procedures then in place. In addition, we provided comprehensive information addressing each of the WoS selection criteria. It was the beginning of a lengthy process. Unlike the evaluation systems used by the National Library of Medicine and Scopus—both of which provide clear timelines and typically complete reviews within 7–9 months—the WoS evaluation procedure does not specify the timing of its steps, leaving journals without updates on their status for months.

**Figure 3:**
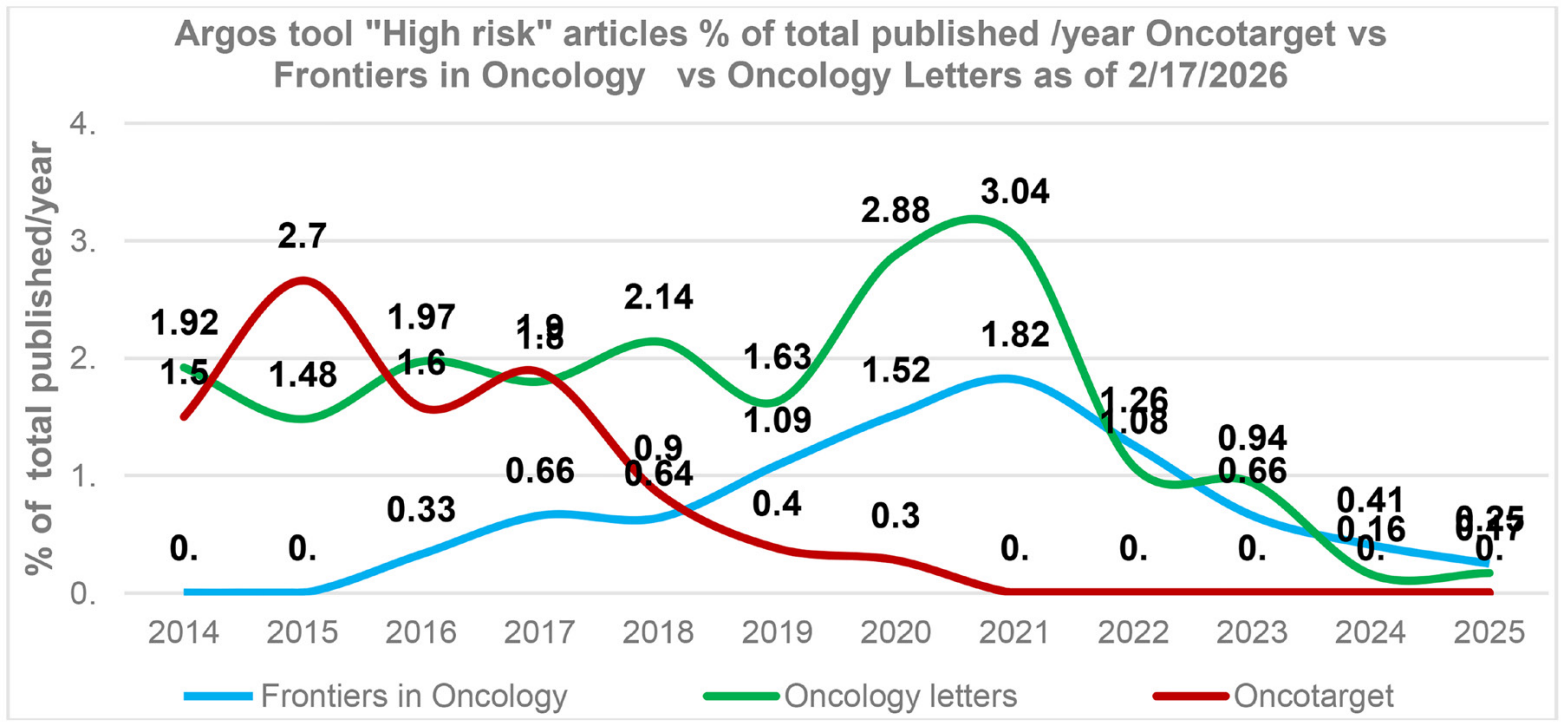
Post-tools era. Oncotarget versus cancer research journals Oncology Letters, Frontiers in Oncology. The percentage of “High risk” articles identified by the tool “Argos” (as of 2/17/2026) in relation to the total number of articles published in a journal during a specific year. The average annual article counts for Oncology letters – 1,226 articles/year, Oncotarget – 2,240 articles/year, and Frontiers in Oncology – 2,885 articles/year.

We also compared cancer research journals with comparable publication volumes. As can be seen in Figure 3, Oncotarget has significantly reduced the number of high-risk papers in the last five years. Frontiers in Oncology also reduced the number of high-risk papers recently. Oncology Letters demonstrates mixed results: while the overall trend shows a decline in high-risk papers, the 2025 data suggests a renewed increase (Figure 3).

It is also of interest to compare Oncotarget with large-scale journals such as Scientific Reports (Nature Portfolio) and PLOS One (the Public Library of Science).

**Figure 4:**
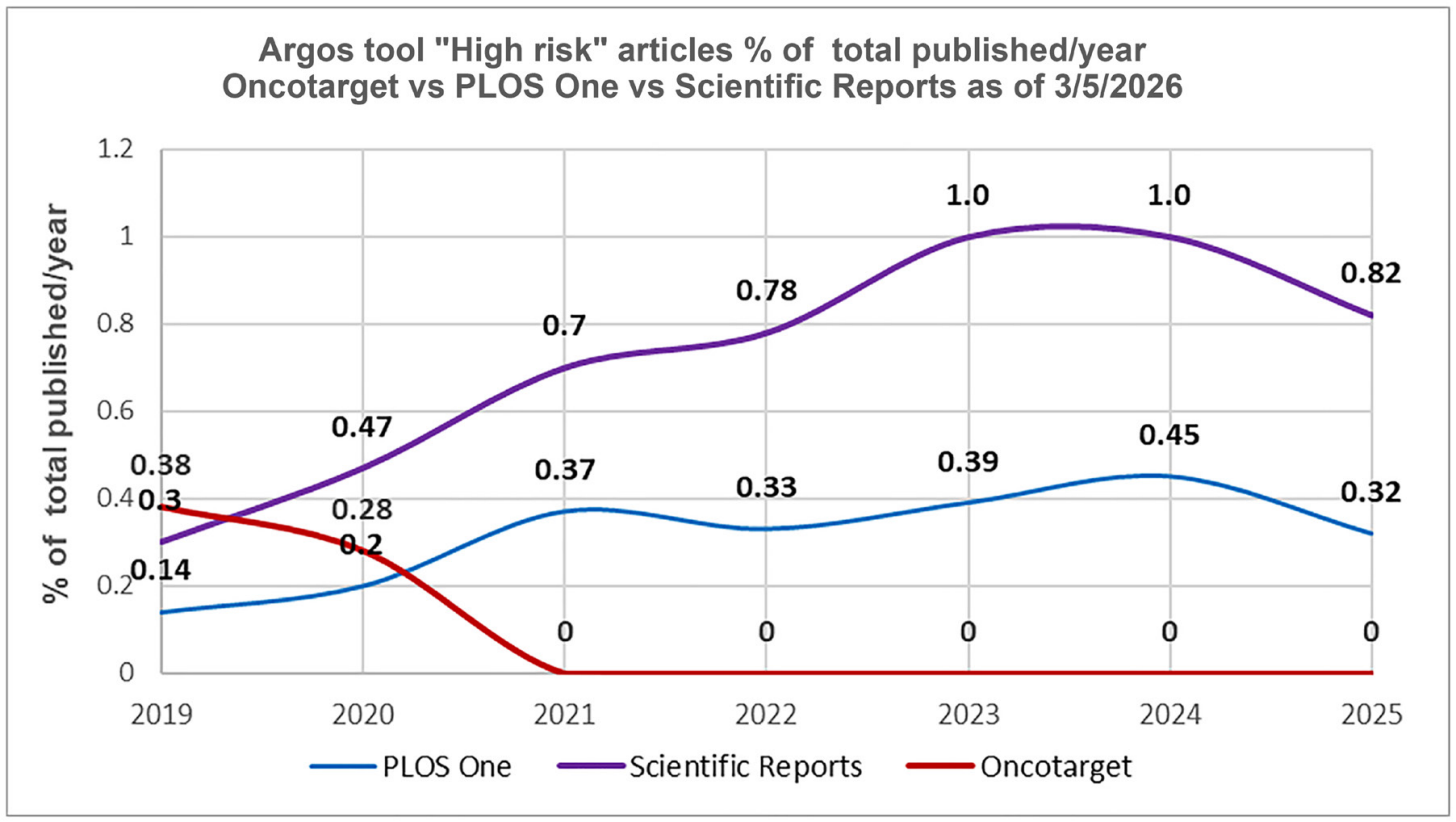
Post-tools era. Oncotarget versus Scientific Reports and PLOS One. The percentage of “High risk” articles identified by the tool “Argos” (as of 3/05/2026) in relation to the total number of articles published in a journal during a specific year in 2019–2025.

Both journals demonstrate an increasing trend of high-risk papers volume from 2019 through 2024 (Figure 4). According to Argos (April 6, 2026), in 2024–2026, *Scientific Reports* is at the top (3rd place) of the list of large journals (more than 10K published papers) with the highest number of “high-risk” articles per 10K published papers. PLOS One ranks eleventh. The list includes 49 journals.

In October 2024, we discussed the Argos data with Features Editor at *Nature*, Richard Van Noorden, and proposed conducting an analysis of different journals and publishers in the “post-tool era”. He agreed that it is an interesting subject: “Regarding: how integrity tools are now changing the publishing industry. I am interested in this topic! At the moment I know I have too much work on to devote time to it, but perhaps could look again in the new year? “In August 2025, during communication with Miryam Naddaf, the *Nature* reporter, we presented the same data and asked for her feedback and opinion. As of today, no response has been received in either case, and it appears that *Nature* has limited interest in pursuing this discussion. We are left to wonder why.

Today, independent analytical tools make it possible to objectively compare different journals. Sometimes such comparison raises questions about a disproportionate coverage of different journals with similar image-related issues, which may result in selective targeting. These problems do exist in the publishing industry and require further analysis and investigation.

**It is time to more broadly use analytical and AI - based tools from independent sources to objectively evaluate the journals and publishing trends in the pre- and post-tools era.**

**It is time for indexes to more broadly adopt independent analytical tools in journals evaluation. Given the position that indexes hold in global scholarly publishing, transparency, consistency, and the absence of double standards in review and indexing policies are essential. Accordingly, open discussion of how indexes select, deselect and reevaluate journals is in the public interest.**

This report measurably demonstrates Oncotarget’s position in addressing scientific integrity. Moreover, our forward-looking strategy is to continue to explore new digital technologies and innovative approaches supporting scientific integrity. We are currently in discussions with several companies that provide such services. Our goal is to ensure that every article we publish continues to meet the most rigorous standards of scientific integrity.

